# Rationale and study design of a randomized, placebo-controlled, double-blind phase 2b trial to evaluate efficacy, safety, and tolerability of an oral glutaminyl cyclase inhibitor varoglutamstat (PQ912) in study participants with MCI and mild AD—VIVIAD

**DOI:** 10.1186/s13195-021-00882-9

**Published:** 2021-08-23

**Authors:** E. G. B. Vijverberg, T. M. Axelsen, A. R. Bihlet, K. Henriksen, F. Weber, K. Fuchs, J. E. Harrison, K. Kühn-Wache, P. Alexandersen, N. D. Prins, Philip Scheltens

**Affiliations:** 1 Alzheimer Center Amsterdam, Department of Neurology, Amsterdam Neuroscience, Vrije Universiteit Amsterdam, Amsterdam UMC, Amsterdam, The Netherlands; 2 Brain Research Center, Amsterdam, The Netherlands; 3 Department of Biomedical Sciences, Faculty of Health and Medical Sciences, University of Copenhagen, Copenhagen, Denmark; 4 Sanos Clinic A/S, Vejle, Denmark; 5 NBCD A/S, Herlev, Denmark; 6 Vivoryon Therapeutics NV, Halle, Germany; 7 Metis Cognition Ltd, Park House, Kilmington Common, Wiltshire, UK; 8 Institute of Psychiatry, Psychology & Neuroscience, King’s College London, London, UK; 9 Alzheimercentrum Amsterdam, Amsterdam UMC, Locatie VUmc, De Boelelaan 1117/1118, 1091 HZ Amsterdam, The Netherlands

**Keywords:** Alzheimer, Clinical trials; Small molecules; Puroglutamate, Abeta, Placebo, Amyloid

## Abstract

**Background:**

Varoglutamstat (formerly PQ912) is a small molecule that inhibits the activity of the glutaminyl cyclase to reduce the level of pyroglutamate-A-beta (pGluAB42). Recent studies confirm that pGluAB42 is a particular amyloid form that is highly synaptotoxic and plays a significant role in the development of AD.

**Methods:**

This paper describes the design and methodology behind the phase 2b VIVIAD-trial in AD. The aim of this study is to evaluate varoglutamstat in a state-of-the-art designed, placebo-controlled, double-blind, randomized clinical trial for safety and tolerability, efficacy on cognition, and effects on brain activity and AD biomarkers. In addition to its main purpose, the trial will explore potential associations between novel and established biomarkers and their individual and composite relation to disease characteristics.

**Results:**

To be expected early 2023

**Conclusion:**

This state of the art phase 2b study will yield important results for the field with respect to trial methodology and for the treatment of AD with a small molecule directed against pyroglutamate-A-beta.

**Trial registration:**

ClinicalTrials.gov Identifier: NCT04498650

## Introduction

Alzheimer’s disease (AD) affects approximately 47 million people worldwide and the prevalence will triple by 2050 [1]. Drugs to halt or slow down the disease process are badly needed. Inadequately designed phase 2 studies have often led to suboptimally designed phase 3 programs and contributed to the high phase 3 failure rate in the past. Dilligently designed phase 2 studies including a broad spectrum of AD-relevant efficacy, biomarker, and safety endpoints are needed to de-risk phase 3 development and lead to a more successful and efficient conduct of development programs with less patients being randomized to or included into large studies with no meaningful outcome.

After the accelerated approval of aducanumab by the FDA on June 7, 2021, the AD field is even more motivated to develop new therapeutic targets associated with amyloid beta aggregation in the brain as well as other possible disease modifying targets [2].

In recent years, it has become clear that the concentration of soluble β-amyloid (AB)-oligomers is more closely associated with clinical symptoms than amyloid plaque burden itself [3–5], by causing more synaptotoxicity, neuroinflammation, and neurodegeneration. Pyroglutamate-AB42 (pGluAB42), a particular AB form, has been found to be extremely toxic, resistant to degradation, more hydrophobic leading to easy aggregation, and seeding the formation of additional neurotoxic oligomers [6–11]. pGluAB42 is generated from full-length AB by various peptidases (e.g., neprilysin (NEP) [12] and insulin-degrading enzyme (IDE) [13]) as part of the normal AB-degradation pathway and are then further modified by the enzyme glutaminyl cyclase (QC) [14]. In human autopsy studies on AD patients, pGluAB42 was found to constitute 10–50% of the total amyloid burden, but not found in plaques of subjects without signs or symptoms of AD, suggesting a significant role in the development of AD [15, 16], consequently making the processes involved with pGluAB42 an interesting therapeutic target, highlighted by the recent encouraging news regarding donanemab, a monoclonal antibody also targeting pGluAB42 [17].

The small molecule QC-inhibitor varoglutamstat inhibits the activity of QC which leads to a reduction of the level of pGluAB42. The dose-dependent inhibition of th QC was demonstrated in a large phase 1 program evaluating up to 14 days of treatment in healthy young and aging individuals being treated with up to 1800 mg of varoglutamstat twice daily (BID). The study found varoglutamstat to be well tolerated with few and mild adverse events (AE) and demonstrated clear pharmacodynamic effects in terms of QC-inhibition in plasma and CSF [18].

In a phase 2a, randomized, double-blind, placebo-controlled, proof of concept trial in biomarker confirmed early AD patients (*n = 120*), study participants were treated with varoglutamstat in doses of 800 mg twice daily for 12 weeks to further evaluate safety and effects on biomarkers [19]. Varoglutamstat showed an acceptable safety and tolerability profile in lower doses and with slower titration. The varoglutamstat-treatment group was also found to have a significant improvement of working memory, reduction of synaptotoxicity measured by less theta-wave activity on EEG, and reduced neurogranin levels as well as improvements on various other experimental biomarkers [19]. Post hoc results further supported the concept of enhance synaptoplasticity with the treatment of varoglutamstat in a network analysis.

The trials with varoglutamstat indicated a beneficial safety and tolerability profle resulting in favorable benefit-risk ratio, biological effect on QC inhibition, reduced synaptic toxicity, data suggestive of a clinical effect, and provided the rationale to design a state-of-the-art phase 2b trial with endpoints in cognitive function, biomarkers, and long-term safety and tolerability. In the following, we describe the study design of the VIVIAD trial, a study to evaluate safety and tolerability of different doses and efficacy of varoglutamstat in study participants with MCI and Mild AD.

## Methods: study setting and design

This multinational, double-blind, randomized, placebo-controlled study is evaluating the disease modifying effects of PQ912 and serves as a dose finding study on the drug in patients suffering from MCI or mild dementia due to AD. The study aims to enroll 250 patients and is currently enrolling study participants in all participating centers.

**Fig. 1:**
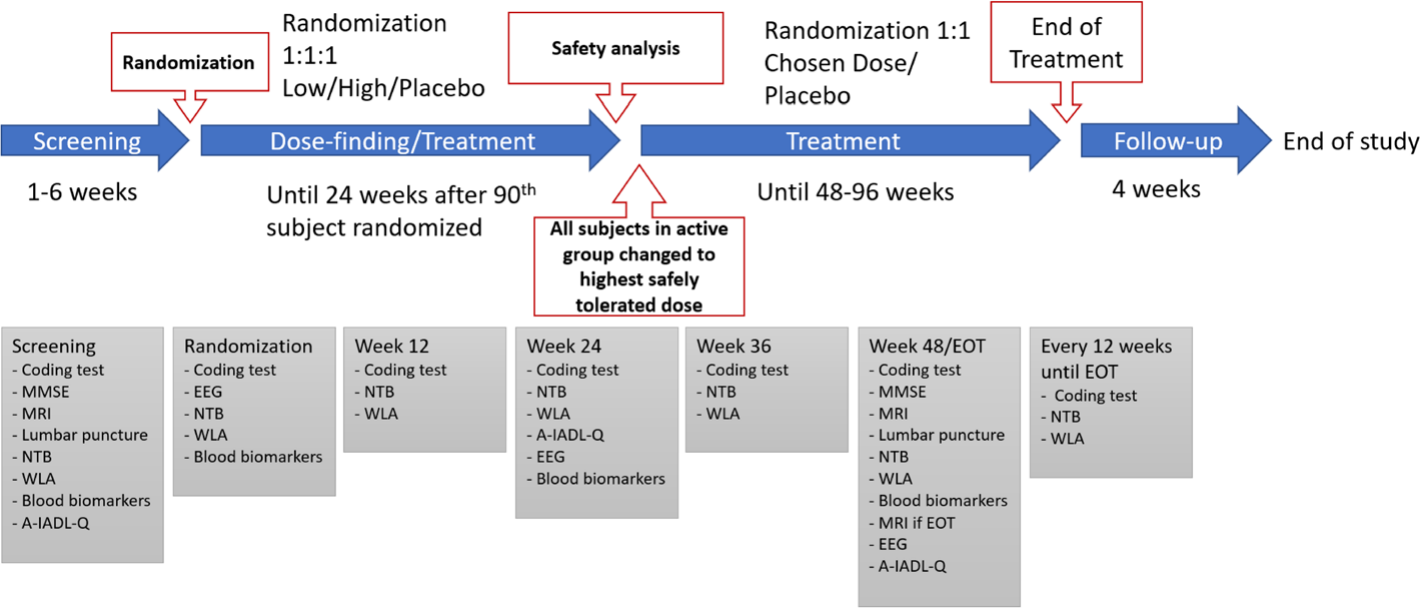
Overview of study design. Coding test, WAIS-IV coding test; MMSE, Mini Mental State Examination; NTB, Neuropsychological Test Battery; WLA, Winterlight Language Assessment; A-IADL-Q, Amsterdam Instrumental of Activities of Daily Living Questionnaire

The trial is designed in two parts which are seamlessly integrated with part one serving as an initial dose-finding phase and part two as a dose confirmation phase. Initially, all participants enrolled will be randomized in a 1:1:1 fashion between 600 mg BID varoglutamstat, 300 mg BID varoglutamstat, or placebo BID. When the 90th subject has completed the evaluation at week 24, an unblinded safety review will be conducted by an independent data safety review board (DSMB) in order to establish the highest well-tolerated dose of varoglutamstat (recommended phase 2b dose). Hereafter, all participants receiving varoglutamstat will be changed to the recommended phase 2b dose, and all further randomization will be done 1:1 between the chosen dose of varoglutamstat and placebo. Study participants recruited early into the study will be kept on treatment for up to 96 weeks or until the last subject recruited will have completed visit (V) 8 (48 weeks of treatment), whichever comes first (see Fig. 1 for graphic overview of the study design). The trial is registered at ClinicalTrials.gov: NCT04498650.

### Study population

The study’s inclusion criteria are study participants, male or female, from ≥ 50 to ≤ 80 years with a diagnosis of MCI or mild dementia due to AD as per the 2018 NIAA research framework [20, 21], with a Mini Mental State Examination (MMSE) score of ≥ 20, and a Wechsler Adult Intelligence Scale (WAIS) IV coding test score [22, 23] ≤ 0.5 standard deviations below the reference score adjusted for age. Our purpose in selecting the coding test requirement was to include only study participants likely to to have a rescuable cognitive deficit. Also, the participants must be able to have a study partner accompany them at study visits, as well as be in a stable state regarding both AD and potential AD-medication.

Exclusion criteria include exclusion of conditions which may affect cognition, including vascular dementia or other cerebral lesions as evaluated by magnetic resonance imaging. No major psychiatric conditions are allowed; there should be no known presence of insufficiently treated vitamin B, folate deficiency, or hypothyroidism. There should be no signs of severe hepatic or renal failure, neither should the participants have suffered stroke or have evidence of other forms of dementia including atypical presentations of AD (e.g., posterior cortical atrophy, frontal or language types of AD) or previous seizures. Patients having had cancer (besides from non-metastatic basal cell cancer or non-metastatic squamous cell cancer) within 2 years, myocardial infarction within the past 6 months, or signs of addiction disorder are also excluded (see Table 1).

**Table 1:** Inclusion and exclusion criteria

• Principal inclusion criteria - Male or female, aged ≥ 50 to ≤ 80 years - A biomarker profile reflecting AD, according to the Alzheimer Association—National Institute on Aging (AA-NIA) Research Framework (Jack et al. 2018) defined as follows: o *a1)* Screening CSF sample with an Aβ42 concentration of < 1000 pg/ml AND p-tau > 19 pg/ml, OR o *a2)* A ratio of p-tau/Aβ42 of ≥0.024 as assessed by central laboratory, (Elecsys assay), OR, in case of study participants in whom CSF sampling is not feasible due to medical or technical reasons. o *b)* Existing positive amyloid positron-emission tomography (PET) evidence within 6 months of the screening visit - Clinical syndrome of MCI or mild dementia according to the AA-NIA Research Framework (Jack et al. 2018) - Signed and dated written informed consent obtained from the subject in accordance with local regulations - A cognitive impairment in the digital symbol substitution test (DSST ) of at least one standard deviation below the normative data - Being in a stable condition with respect to the current AD condition: either without specific treatment or in a stable dose of AD medication for the past 10 weeks - Outpatient with study partner capable of accompanying the subject on screening visits, week 24, 48 weeks, and at the end of treatment visit (EOT) Principal exclusion criteria - Significant neurological or psychiatric disorders other than AD that may affect cognition - Atypical prenstations of MCI due to AD or mild dementia due to AD (such as posterior cortical atrophy, frontal variant, or language variant) - Moderate and severe dementia with a MMSE below 20 - History of (maximally 6 months from screening) or screening visit brain MRI scan indicative of any other significant abnormality, including but not limited to severe white matter hyperintensities (Fazekas score 3), history or evidence of a single prior hemorrhage > 1 cm^3^, multiple lacunar infarcts or evidence of a single prior infarct > 1 cm^3^, evidence of a cerebral contusion encephalomalacia, aneurysms, vascular malformations, subdural hematoma, or space-occupying lesions (e.g., brain tumors) - Current presence of a clinically important major psychiatric disorder (e.g., major depressive - disorder) as defined by DSM-5 criteria, or symptom(s) (e.g., hallucinations) that could affect the subject’s ability to complete the study - History of stroke or seizures - Myocardial infarction within 6 months of screening - History of cancer within the past 2 years prior to screening (except: non-metastatic basal cell carcinoma, and squamous cell carcinoma of the skin) OR no signs of residual cancer confirmed minimum 6 months before baseline - History of uncontrolled hypertension within 6 months prior to baseline - Hemoglobin level less than 11 g/dL at screening - Clinically important infections within 30 days prior to screening - Insufficiently or untreated hypothyroidism, B_12_ or folate deficiency - Severe hepatic failure (Child-Pugh C) or kidney failure (creatinine clearance (eGFR) ≤ 30 mL/min/1.73 m^2^) as estimated using the MDRD method, or serum creatinine above 1.5-fold of upper limit of normal (ULN) or asparagine-amino transferase (AST) or alanine-amino transferase (ALT) above 3-fold of ULN at screening.	

As for prohibited medication, varoglutamstat is a moderate inhibitor of CYP2C19; therefore, concomitant administration with strong inhibitors of the CYP2C19 like fluconazole, fluvoxamine, and ticlopidine or moderate inducers of CYP2C19 (rifampicin) and with CYP2C19 substrates with a narrow therapeutic margin like S-mephenytoin, phenytoin, phenobarbital, and indometacin should be avoided and replaced by alternative products before starting PQ912 exposure.

## Results: study procedures

The screening for the trial consists of several elements in order to ensure adherence to the inclusion and exclusion criteria (see Table 1). Prior to or at the study baseline visit, the medical history, physical and neurological examination, vital signs, ECG, and cognitive measures including the WAIS-IV coding test, to test executive function and working memory, and the MMSE will be assessed. A cerebral MRI will be performed to rule out relevant cerebral vascular disease or other pathological changes. A lumbar puncture will be performed to assess inclusion criteria in the form of AB42 and p-Tau-levels with an Aβ42 concentration of < 1000 pg/ml AND p-tau > 19 pg/ml or a ratio of p-tau/Aβ42 of ≥ 0.024 (Elecsys assay) to be noted as abnormal. The laboratory assessments include standard parameter for hematology, biochemistry, apolipoprotein E (ApoE), human leukocyte antigen (HLA), and CYP2c19 genotyping.

Additionally, prior to randomization, the baseline values for the primary, secondary, and exploratory efficacy endpoints will be recorded including a Neuropsychological Test Battery (NTB) [24–29] composed of CogState tests of episodic verbal memory (the International Shopping List Test), episodic visual memory (the One Card Learning test), working memory (the One Back test), and two measures of attention (“Detection” and “Identification”). As a means of evaluating activities of daily living in this cognitively impaired patient population, the Amsterdam Instrumental of Activities of Daily Living Questionnaire (A-IADL-Q) [30, 31] will be employed. This instrument is completed by the study partner; hence, study participants are required to have a study partner able to accompany them on visits to clinic. To assess AD-specific speech changes, the Winterlight speech assessment (WLA) will be assessed at baseline as it will be employed as an exploratory endpoint in the study [32].

Upon passing screening, the first 90 participants will be randomized 1:1:1 by a computerized allocation system to receive either PQ912 300 mg BID, 600 mg BID, or placebo BID following a 12-week escalation period. Escalation occurs in up to five steps: weeks 1–2: 50 mg/placebo once daily, weeks 3–4: 50 mg/placebo BID, weeks 5–8: 150 mg/placebo BID, weeks 9–12: 300 mg/placebo BID, and weeks 13–24: 300 mg/placebo or 600 mg/placebo BID, in order to minimize possible side effects.

As illustrated in Fig. 1, the participants will be seen in the clinics 4 weeks after baseline and assessed for AEs, drug accountability, and vital signs. In addition, blood will be sampled for hematology and biochemistry, and a physical exam will be conducted. At week 12, the above procedures will be repeated as well as the NTB, WLA, and WAIS-IV coding test. These procedures will be repeated every 12 weeks.

The participants attend clinic visits once every 12 weeks until study termination, where several key measurements are made at timepoints, including physical and neurological examination, WAIS-IV coding test, WLA, NTB, and Amsterdam IADL-Q. Every 24 weeks, the blood will be analyzed for biomarkers. Blood levels of PQ912 will be measured at weeks 12, 24, and 48 as well as at EOT, for study participants enrolled early in the trial. In addition, an EEG will be performed at weeks 24 and 48, and an additional cerebral MRI will be performed at EOT for safety. CSF will be collected for biomarker measurement at week 48.

Should the patients experience significant intolerable side effects (starting from a maximal dose of 300 mg BID/placebo or above), the investigators may decrease drug dosage by 50% for a period or temporarily suspend treatment with IMP. Should treatment-naïve study participants progress significantly in their symptoms during the study, initiation of standard AD medication is allowed.

### Endpoints

The safety analysis will be based on significant changes on (i) physical and neurological examinations, (ii) significant changes on blood samples, (iii) amyloid-related imaging abnormalities (ARIA), and (iv) spontaneously reported adverse events.

The primary efficacy analysis consists of the pooled Z-score of the CogState “Detection,” “One Back,” and “Identification” tests (see the “Statistical evaluation” section for method of evaluation).

In addition to the primary analysis of efficacy, several secondary and exploratory efficacy endpoints have been defined for this study: the linear change in overall cognition based on the complete NTB and the Brief CogState battery and baseline to week 48 changes in global relative theta power (4–8 Hz) in the EEG-data as well as of the A-IADL-Q.

Above the use of traditional measures of cognitive assessment, we have also incorporated speech analysis into the trial protocol. While the use of this technology is still largely experimental, it offers a reliable and valid assessment of the speech production difficulties in common individuals living with AD. The reliance on subjective impressions of speech performance, such as those included in the ADAS-cog, has likely underestimated the frequency and magnitude of these difficulties. The use of technology such as the WLA system offers the opportunity to assess speech difficulties objectively.

As an exploratory endpoint, neuronal activity will be evaluated from EEG recordings in the same manner as described by Briels et al. [32, 33], Poil et al. [34], and Scheltens et al. [19] as regards the global relative power in the delta (0.5–4 Hz), alpha (8–13 Hz), and beta (13–30 Hz) frequency bands, looking into global posterior dominant peak frequency, amplitude envelope correlation (AEC) in the 4–13 Hz band, and functional network topology measures such as centrality, modularity, and minimum spanning tree in an attempt to acquire new EEG AD-specific biomarkers in this modality.

Blood-based biomarker will be measured at baseline, weeks 24 and 48, and the EOT visit, and CSF-based biomarker is measured at screening and at week 48. There will be a broad panel of biomarkers assessed with potential use for quantifying neuroinflammation, synaptic toxicity, and neurodegeneration. Individual parameter assessed will include levels of the following: YKL-40 (inflammatory marker), neurogranin (synaptic marker), beta secretase 1 protein [BACE-1], Tau and pTau, protein fragments of Tau, GFAP, and extracellular matrix (ECM) molecules (neurocan, brevican, and Tenascin-R). In addition to the earlier mentioned well-established biomarkers, exploratory biomarkers in both plasma and CSF will be investigated, e.g., neurofilament light chain (NFL); protein fragments of Tau, GFAP, and extracellular matrix (ECM) molecules (neurocan, brevican, and Tenascin-R); pGlu-peptide substrates of QC (e.g., pGlu- and total C-C motif chemokine ligand 2 [CCL2], Orexin A), and Aβ peptides (including full length and truncated A peptides) will be assessed.

### Statistical evaluation

The main statistical evaluation will be performed in the full analysis set population, such that all participants who are randomized and received at least one dose of study medication will be included in the main statistical analysis.

An unblinded safety review is planned to be performed shortly following the 90th participant reach 24 weeks post-baseline. An independent data safety monitoring board (DSMB) consisting of experts with relevant medical and scientific expertise will conduct an unblinded safety analysis, in order to decide—based solely on the safety profile of the 300 and 600 mg dose groups—which dose level is recommended to be carried forward into the phase 2b part of the study. The DSMB may likewise decide to discontinue the study at this—and at any other timepoint during the trial—if the safety profile of the investigated dose levels of varoglutamstat is not acceptable. No efficacy data will be assessed for deciding which dose level will be carried forward because the efficacy data are too immature at this timepoint to contribute to the decision.

**Fig. 2:**
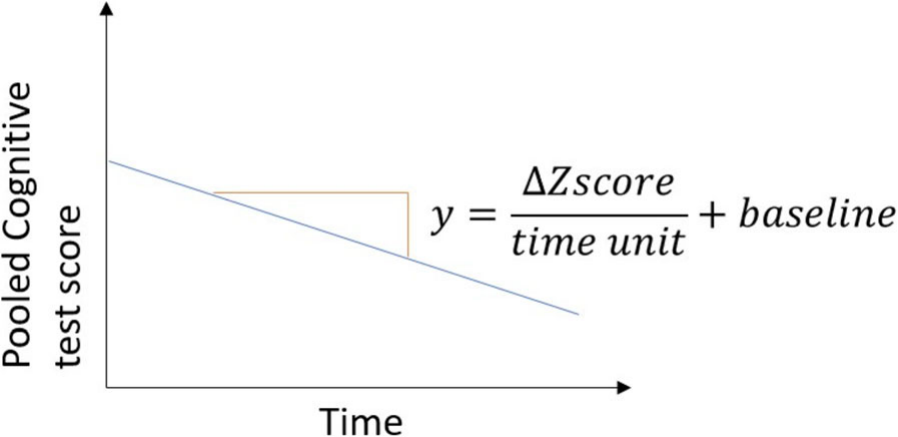
Conceptual figure denoting statistical approach to incorporate the variable treatment times of the enrolled study participants in the data analysis

As the study is planned to have differences in participation duration and dosage, a linear model of the primary efficacy parameter will be employed to assess effectiveness. The primary efficacy analysis will consist of the pooled *Z* score based on the cognitive scores from the CogState “One Back,” “Identification,” and “Detection” tests (see Fig. 2). In the analysis, all patients will be included independently whether they were initially on a different dose than the one carried forward after the DSMB recommendation. This method is consdiered acceptable as both doses lead to a a QC inhibition in a similar range (70–85%). It is assumed that the *Z* score will change linearly with time both in the actively treated and the placebo group. The treatment effect of the primary efficacy measure will be assessed in a linear mixed model with random coefficients. In this model, the treatment effect will be estimated as the difference in slopes between the active and the placebo arms. This approach enables incorporation of all data on participating individuals, irrespective of their follow-up time, thus ensuring a maximum amount of information obtained from study participants over the course of the trial.

Based on extrapolation of the results in the SAPHIR study, evaluation of other empirical data, and expert opinion, it was determined that at 48 weeks, an effect size of a Cohen’s *d* of approximately 0.35 is a reasonable expectation. This effect sizecorresponds to a marked reduction of the decline in the combined *Z* score for cognition in the PQ912-treated study participants and a linear progression in the placebo-treated patients. Based on the assumptions above, and an allocation ratio of 11:14 between active and placebo, by comparison via a two-sample *T* test, a total sample size of around 250 should provide a power close to or exceeding 80%.

The sample size is not adjusted for the rate of drop-out as it is anticipated that all participating individuals will have at least two measurements of the combined *Z* score, irrespective of their follow-up time, and therefore contribute to the analysis of the primary efficacy endpoint.

## Discussion

The VIVIAD trial is a phase 2b dose-finding study further evaluating the safety and efficacy of varoglutamstat, as a potential disease-modifying treatment in AD. Varoglutamstat inhibits the QC activity and reduces the level of pGluAB42, which is expected to alleviate the acute and chronic neurotoxic effects of pGluAB42. In addition to its main purpose, the trial will explore potential associations between novel and established biomarkers and their individual and composite relation to disease characteristics.

Dose finding for the optimal benefit-risk ratio is an important part of the VIVIAD trial. The previous 2a study established 800 mg varoglutamstat BID to be the maximum tolerable dose. It is expected that a slower dose titration may improve safety and tolerability. The rationale behind a gradual titration is that most AEs from the SAPHIR trial was deemed of immunological origin [19]; a titration period may allow a gradual habituation of the immune system to the drug. So, the current trial has a gradual dose escalation period, followed with an interim analysis in order to establish the most suitable dose of varoglutamstat while limiting the risk of potential negative consequences of QC inhibition in the periphery for the rest of the trial.

One of the primary efficacy endpoints is the evaluation of varoglutamstat on working memory and attention, and secondarily on cognition and activities of daily living, using the Cogstate Neuropsychological Test Battery and the A-IADL-Q, both of which have been validated for use as serial measurements [24, 25, 30]. Exploratory measures include the WAIS-IV coding test [22, 35], part of the WAIS-IV intelligence test which has also been validated for exploring executive function and working memory as a stand-alone test and which is recognized by the EMA as a measure of “timed executive function.” The WAIS-IV coding test serves in this study as an inclusion criteria with a specific cut-off in the screening process, selecting only participants with a certain degree of cognitive impairment. Using the WAIS-IV coding test score as a marker (longitudinal evaluation) is a novel approach in AD clinical trials.

Results from SAPHIR trial indicated beneficial effects of varoglutamstat on synaptic toxicity measured by different biomarkers. Therefore, the efficacy of varoglutamstat on brain activity will be assessed as a secondary objective. Based on the positive findings in the SAPHIR study [19, 33], resting-state brain activity will be evaluated by using EEG which has been found to be associated with synaptic activity enabling a macroscale assessment of neuronal circuit integrity [33, 36]. Besides that, EEG is both cost-effective and widely available, making it very suitable for trials in AD due to the method’s ability to detect early changes in oscillatory activity of the cortex [21]. Another argument for including EEG changes as an endpoint is that the functional neurophysiological changes of a treatment can happen within days to weeks, as shown in the classical cholinesterase studies. In the VIVIAD trial, EEG data will be assessed for changes in theta-wave activity (theta power) and other sensitive oscillatory activity markers [23, 31]. Incorporation of this modality in the current study may further validate EEG changes as endpoints in clinical trials.

Another novel outcome in the VIVIAD trial is an automated speech recognition method-based measure of disease affecting language (including AD), the WLA. Most individuals living with AD exhibit some degree of speech impairment [27]. This worsens over time [28, 29], making speech and language assessments relevant for appraisal of disease severity and activity. However, changes over time of each individual’s language and in early stages of AD can be subtle and difficult to detect. Previous speech evaluation has relied on cumbersome tests unsuitable for widespread clinical use [37]. By employing an algorithm evaluating factors such as length of sentences, richness of vocabulary, repetitiveness, and spectrum of acoustics, it is possible to extract accurate data in speech passages as brief as 150 words. Digital speech biomarkers have, so far, only been used in an experimental diagnostic setting [26, 38, 39]. The longitudinal setting of this trial allows for analysis of the WLA as an exploratory endpoint, as well as a tertiary measurement of efficacy of varoglutamstat.

Changes in biochemical biomarkers are important outcome measurements to detect biological/pharmacodynamic effect in phase 2b AD trials. In the SAPHIR trial [19], YKL-40, a neuroinflammation marker in AD [40], was shown to decrease approximately 5% from baseline levels within 12 weeks when using varoglutamstat. Additionally, the synaptic biomarker neurogranin was reduced by approximately 4%. Both of these biological signals were meaningful when considered in relation of increase in general AD patients. In the current trial, biochemcial biomarkers serve several purposes: they act as an inclusion criterion, minimizing the risk of including non-AD patients into the trial and also function as outcome measures on week 48/and or end of treatment. Serum and CSF will be assessed for QC activity, inflammatory, synaptic, neurodegeneration, and AD biomarkers.

The statistical approach to the primary and some of the secondary endpoints allows for interpretation of the effect of varoglutamstat in a larger sample of individuals, as it does not require all study participants to be evaluated at a specific time point. This is highly beneficial, as it allows inclusion of the data from the dose-finding phase into the primary endpoint analysis. Based on the phase 1 trial, the difference between 600 mg BID and 300 mg BID in CSF QC-inhibition will be approximately 10% [18]; whether this difference is clinically significant is unknown, but as AB is a poor substrate of glutaminyl cyclase, smaller differences of reductions in QC activity are not expected to change the inhibition of the formation of neurotoxic pyro-GluAB. In addition, the inclusion of the dose finding phase in the data analysis allows a longer follow-up of the treated individuals and provide information on the long-term treatment effect regarding both safety and efficacy data without affecting the time to result.

In conclusion, the present VIVIAD trial is a state-of-the-art design in the field of AD and is a combined dose-finding, proof-of-concept efficacy and safety analysis of varoglutamstat with a strong exploration platform for the development of new important biomarker-based diagnostics in one and the same trial.

## Data Availability

n/a
